# Acute severe non-traumatic muscle injury following reperfusion surgery for acute aortic occlusion: case report

**DOI:** 10.1186/1865-1380-4-20

**Published:** 2011-04-28

**Authors:** Joseph Y Ting, Arash Dehdary

**Affiliations:** 1 Department of Emergency Medicine, Mater Public Hospitals, South Brisbane 4101, Australia

## Abstract

Acute aortic occlusion is a rare but catastrophic disease with a high mortality rate. Severe perioperative complications could result from revascularization of infarcted muscles. Muscle cell ischaemia and massive volume cell death lead to the release of myoglobin, potassium, and lactic acid, which could be fatal if not recognised or treated early. We highlight the life-threatening adverse effects resulting from bulk tissue infarction from non-traumatic causes such as aortic occlusion followed by the metabolic sequelae of reperfusion. This is similar to the pathophysiology of traumatic crush injuries and rhabdomyolysis. The case highlights the vigorous pre-emptive treatment of acidosis and hyperkalaemia required during surgical revascularisation to potentially avert adverse surgical outcomes in acute aortic obstruction.

## Background

Acute aortic occlusion is a rarely encountered but frequently fatal emergency, resulting from de novo thrombus formation subjacent to atherosclerotic aortic mucosal lining or the peripheral embolisation of dislodged centrally located thrombus to obstruct a previously healthy aorta.

We describe the de novo hyperacute development of totally occlusive extensive infrarenal aortic thrombus that progressed to bilateral limb-threatening ischaemia that on surgical reperfusion led to a metabolic surge (acidaemia, hyperkalaemia) that eventuated in irreversible cardiac arrest. The case highlights the need to anticipate early, and pre-emptively treat, life-threatening toxic metabolic surge from acute compartment release and reperfusion of non-traumatic bulk tissue infarction, a risk well recognised in rhabdomyolysis from traumatic crush injury.

## Case presentation

A 73-year-old man presented to the emergency department with sudden epigastric and bilateral flank pain after vomiting. Pain and paresthesia worsened rapidly in the lower trunk and both legs within an hour of arrival.

The patient had a history of extensive peripheral vascular disease, having had a left iliac artery thrombectomy with a femoro-femoral crossover graft 2 years previously. He was being investigated for a paraaortic mass; CT abdomen demonstrated a large mass left of the aorta blending with the underlying psoas and disturbing the aortic outline on the left. The diagnostic possibilities included sealed aortic leak following a local dissection, thrombosed saccular aneurysm, a metastatic mass, or lymphoma. A CT-guided biopsy showed necrotic tissue only. He was on aspirin 100 mg daily and smoked heavily.

The patient was distressed by pain with a pulse of 140/min, blood pressure 172/136 mmHg, SaO2 100% on 8 l/min of oxygen, and temperature of 35°C. The abdomen was diffusely tender, guarded, and rigid. The lower abdomen and both lower limbs felt pale and cold. Femoral, popliteal, posterior tibial, and dorsalis pedis pulses were absent including for Doppler signal. He had symmetrical lower limb complete paresis with areflexia. The lower limb muscle compartments were not firm or tender, and active extension of the knee and ankle did not exacerbate pain.

The first EKG showed broad complex sinus tachycardia. The patient then developed monomorphic VT without altered consciousness level, for which he received IV 50 mg lidocaine followed by 300 mg amiodarone over 1 h. This resulted in reversion to sinus tachycardia at 145/min.

Initial serum urea and electrolytes showed no acidemia or hyperkalaemia: [K^+^] 4.0 mmol/l (3.2-4.5 mmol/l), [Na^+^] 143 mmol/l (135-145mmol/l), and [bicarbonate] 20 mmol/l (22-33 mmol/l). There was a mild coagulopathy-activated partial thromboplastin time of 41.1 s (22-35 s), prothrombin time of 18.8 s (11-16 s), and fibrinogen of 0.9 g/l (1.5-4 g/l). Urinalysis showed no haemoglobinuria suggestive of myoglobinuria.

**Figure 1:**
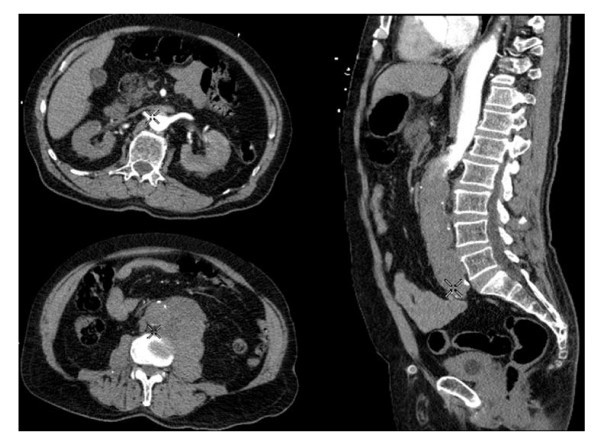
**CT aortography**. An 11.5 × 9 × 6 cm solid mass lying on the left side of the mid-abdominal aorta is shown. Contrast was seen extending to the level of the mass. Coeliac axis, superior mesenteric and renal arteries were filled but no opacification was present through the distal aorta and iliac arteries. Some contrast was seen in distal external iliac arteries, presumably arising from collateral vessels. Both kidneys did not enhance well, suggesting vascular compromise in both.

His presentation suggested ruptured abdominal aortic aneurysm, and an urgent bedside abdominal USS was arranged. This showed a large non-aneurysmal upper abdominal mass related to the aorta. The abdominal aorta could not be identified below the mass, suggesting distal arterial insufficiency at a high level. CT abdomen with intravenous contrast was then performed, demonstrating a solid mass lying to the left of the aorta (Figure 1). Contrast was seen extending to the level of the mass but not below. Arterial phase perfusion of both kidneys was reduced.

ED management included parenteral narcotic analgesia, IV heparin infusion, and IV fluid resuscitation. A bypass procedure to re-establish perfusion was decided upon. The patient underwent axillo-femoral bypass over 90 min. Hypotension (systolic blood pressure <100 mmHg) throughout the intraoperative period did not respond to a noradrenaline infusion.

The first intraoperative arterial blood gas (ABG) was performed close to establishing bypass re-perfusion: pH 7.13 (7.35-7.45), pCO2 51 mmHg (35-45 mmHg), [bicarbonate] 16 mmol/l (22-27 mmol/l), pO2 165 mmHg (70-100 mmHg), [Na^+^] 140 mmol/l, and [K^+^] 4.7 mmol/l. Immediate postoperative ABG showed worsening acidemia and hyperkalaemia (pH 7.025, pCO2 49.6 mmHg, pO2 95.5 mmHg, [bicarbonate] 12.3 mmol/l, [Na^+^] 143 mmol/l, and [K^+^] 6.7 mmol/l), which was treated with 10 mmol intravenous calcium chloride.

**Figure 2:**
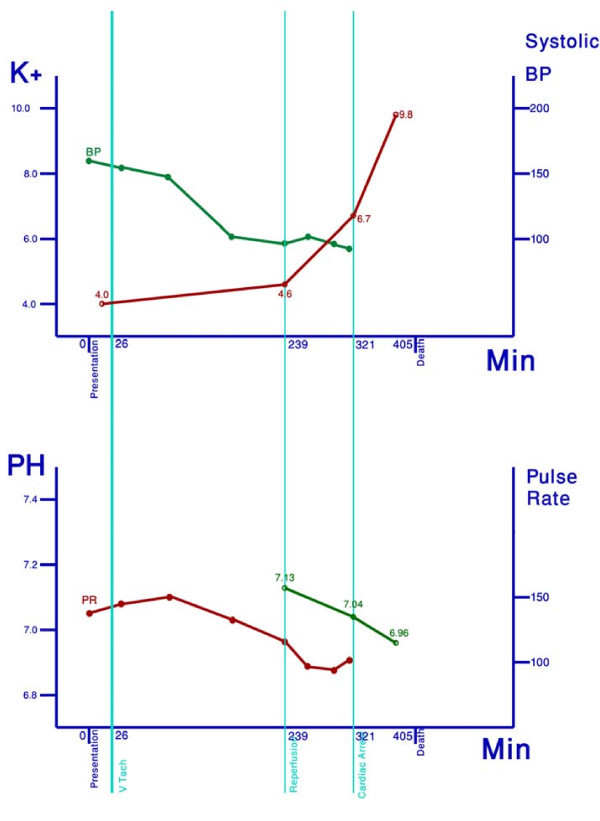
**Time series of vital signs, serum potassium, and arterial pH**. Hyperacute elevation in potassium levels and declining pH preceded sudden perioperative deterioration.

Soon thereafter, the patient suffered a ventricular fibrillation and subsequent asystolic arrest, which did not respond to advanced cardiac life support measures, an IV 50 ml 50% dextrose with 10 units of insulin, and an adrenaline infusion. A third ABG at mid-resuscitation attempt showed non-life-compatible deterioration in acid-base and potassium status pH 6.959, pCO2 37.1 mmHg, pO2 80.1 mmHg, [bicarbonate] 7.9 mmol/L, [Na^+^] 137 mmol/l, and [K^+^] 9.8 mmol/l (not hemolyzed). The time series of perioperative deterioration is demonstrated in Figure 2.

## Conclusions

Acute aortic occlusion is a rare but catastrophic disease, with a high mortality rate of 75%, especially if there are delays in diagnosis and treatment. It results from aortic saddle embolus or the development of acute occlusive thrombosis overlying atherosclerotic abdominal aorta or damaged aortic intima from blunt trauma [1-3]. The embolus usually originates from a thrombus within cardiac chambers resulting from atrial fibrillation, eventually lodging at the aortic bifurcation [2,3].

The presenting signs and symptoms include lower back, buttock, and lower extremity pain, motor and sensory deficit in the lower extremities, absence of palpable pulses in the lower extremities, and mottling from the waist down [1-3]. Severity of symptoms depends on the acuity of onset and time required for collateralization. Patients frequently have coexisting diffuse arterial disease including coronary artery and cerebrovascular disease [1]. The main complications of aortic occlusion include ischaemia of the lower limbs [2], as had occurred in this case.

Prognosis depends on early diagnosis and timely reperfusion of critically ischaemic tissue. Supportive measures include central venous-guided aggressive intravenous hydration to preserve renal function and maintain hemodynamic stability as well as reduction of clot extension with early institution of parenteral anti-coagulation. Surgical revascularization by thrombectomy or bypass provides definitive care [1,3]. The surgical technique used is dependent on the cause, site, and extent of aortic obstruction, patient comorbidities, and clinical condition at presentation [3]. They include direct arteriotomy, passage through the clot of balloon catheters, retrograde flushing, and bypass grafting procedures [2,3]. Aortoiliac occlusion requires bypass procedures, with axillofemoral bypass grafting use indicated in high premorbid surgical risk patients, such as our case [3]. Intraarterial streptokinase could be considered as primary treatment in patients who are not surgical candidates [3].

Perioperative complications could result from revascularization of infarcted muscles. Acute reperfusion of muscle compartments leads to sudden intravascular dissemination of toxic metabolites and potentially acute rhabdomyolytic renal failure. Muscle cell ischaemia and massive volume cell death lead to the release of myoglobin, potassium, and lactic acid, which could be fatal if not recognised or treated early [3].

This case highlights the life-threatening adverse effects resulting from bulk tissue infarction from non-traumatic causes such as aortic occlusion followed by the metabolic sequelae of reperfusion. This could be considered to be similar to the pathophysiology of traumatic crush injuries and rhabdomyolysis. Hypovolaemia as a result of fluid shift into critically ischaemic tissue, acute severe hyperkalaemia, and acidosis are frequently the ultimate cause of perioperative death in patients with aortic occlusion [4]. As in rhabdomyolysis we feel that a vigorous pre-emptive approach including early treatment of acidosis and hyperkalaemia during revascularisation could enhance the outcome of surgery in patients with acute aortic obstruction.

## Consent

This patient has no listed or known family or partner next of kin - his demise was notified to the local police precinct (Annerley, Brisbane).

## List of abbreviations

SaO2: oxygen saturation on pulse oximetry; VT: ventricular tachycardia; [Na^+^]: serum sodium concentration; [K^+^]: serum potassium concentration; USS: ultrasound scan.

## Competing interests

The authors declare that they have no competing interests.

## Authors' contributions

AD wrote the first draft. This and subsequent versions were revised substantially for both form and content by JYT. Both AD and JYT fulfil the three criteria required for authorship.
